# The Prevalence of Acute Respiratory Distress Syndrome (ARDS) and Outcomes in Hospitalized Patients with COVID-19—A Study Based on Data from the Polish National Hospital Register

**DOI:** 10.3390/v14010076

**Published:** 2022-01-01

**Authors:** Mariusz Gujski, Mateusz Jankowski, Daniel Rabczenko, Paweł Goryński, Grzegorz Juszczyk

**Affiliations:** 1 Department of Public Health, Medical University of Warsaw, 02-097 Warsaw, Poland; mariusz.gujski@wum.edu.pl (M.G.); grzegorz.juszczyk@wum.edu.pl (G.J.); 2 School of Public Health, Centre of Postgraduate Medical Education, 01-826 Warsaw, Poland; 3 Department of Population Health Monitoring and Analysis, National Institute of Public Health—National Institute of Hygiene, 00-791 Warsaw, Poland; daniel@pzh.gov.pl (D.R.); pawel@pzh.gov.pl (P.G.)

**Keywords:** SARS-CoV-2, COVID-19, ARDS, ICU, mortality, outcomes, hospitalization, Poland

## Abstract

Acute respiratory distress syndrome (ARDS) is a serious complication of COVID-19. This study aimed to evaluate the prevalence of ARDS among patients hospitalized with COVID-19 in Poland as well as to characterize clinical outcomes in patients hospitalized with COVID-19-associated ARDS. This is a retrospective, secondary analysis of epidemiological data from 116,539 discharge reports on patients hospitalized with COVID-19 in Poland between March and December 2020. The overall prevalence of ARDS was 3.6%, respectively 2.9% among females, and 4.4% among males (*p* < 0.001). Of the 4237 patients hospitalized with COVID-19-associated ARDS, 3764 deaths were reported (88.8%). Participants aged 60 years and over had more than three times higher odds of COVID-19-associated ARDS. Men had higher odds of COVID-19-associated ARDS than women (OR = 1.55; 95% CI: 1.45–1.65; *p* < 0.001). Patients with COVID-19 and diabetes had higher odds of COVID-19-associated ARDS (OR = 1.16; 95% CI: 1.03–1.30; *p* = 0.01). Among patients with COVID-19-associated ARDS, older age, male sex (OR = 1.27; 95% CI: 1.03–1.56; *p* = 0.02), and presence of cardiovascular diseases (OR = 1.26; 95% CI: 1.00–1.59; *p* = 0.048) were significantly associated with the risk of in-hospital death. Among patients hospitalized with COVID-19 in Poland, the prevalence of ARDS was relatively low, but the in-hospital mortality rate in patients with COVID-19-associated ARDS was higher compared to other EU countries.

## 1. Introduction

Coronavirus disease 2019 (COVID-19) is an infectious disease caused by severe acute respiratory syndrome coronavirus 2 (SARS-CoV-2) [1,2]. The median incubation period of COVID-19 from exposure to symptoms onset is 4–5 days [3]. Illness severity can range from mild to critical [4]. Symptoms may differ with the severity of the disease [3,4]. Moreover, asymptomatic infections have been reported [5].

Most cases of COVID-19 are mild or moderate and do not require hospitalization [3,4,5]. Fever, cough, shortness of breath, chills, fatigue, muscle aches, headache, loss of taste or smell, sore throat, rhinorrhea, vomiting, diarrhea are the most common COVID-19 symptoms [4]. Some data suggest that the Delta variant is more contagious and might cause more severe illness than variants observed in the first half of 2020 [6]. Moreover, in November 2021, numerous countries around the world designated Omicron as a variant of concern [7].

It is estimated that 3–5% of COVID-19 cases are severe and require hospitalization [8]. The risk of severe COVID-19 increases with age [8,9]. According to the European Centre for Disease Prevention and Control (ECDC) estimates, 7.5% of patients diagnosed with COVID-19 between August 2020 and May 2021 required hospitalization [9]. The hospitalization rate varied from 1.3% in patients under 30 years to 34.9% among patients aged 80 years and over [9]. In the European Union (EU), the case-fatality rate ranges from 4.0% in Bulgaria to 0.7% in Finland [10].

Acute respiratory distress syndrome (ARDS) is a serious complication of COVID-19 [11,12,13]. Patients with moderate-to-severe ARDS require invasive mechanical ventilation (IMV) and extensive medical treatment [11,13]. Moreover, patients who survive ARDS have reduced exercise capacity and health-related quality of life [12]. A global literature survey on the incidence of ARDS and outcomes in hospitalized patients with COVID-19 showed that approximately 33% had developed ARDS [12]. A meta-analysis of 10,815 ARDS cases in COVID-19 patients from seven countries showed that the overall pooled mortality estimate was 39% [14]. Out of seven analyzed countries, the highest mortality estimate among COVID-19 patients with ARDS was reported in Poland (73%; 95% CI: 58–86%) and the lowest in Germany (13%; 95% CI: 2–29%) [14]. Welker et al. showed that a dysregulated inflammatory response and endothelial thrombosis may be key features differentiating COVID-19 ARDS from traditional ARDS, but the recommended treatment methods include established supportive therapies (lung-protective ventilation and prone positioning) regardless of the type of ARDS (traditional vs. COVID-19-associated) [15]. Bain et al. showed that patients with COVID-19-associated ARDS had significantly higher body mass index and were more likely to be Black or residents of skilled nursing facilities, compared with those with non-COVID-19 ARDS [16]. Moreover, in COVID-19 patients, ARDS was associated with longer dependence on mechanical ventilation compared with patients with traditional ARDS [16]. COVID-19-associated ARDS pathophysiology and treatment guidelines are part of numerous clinical trials around the world [15].

Poland is a country with one of the highest COVID-19 burdens in the EU [8]. As of 15 December 2021, a total of 3,881,348 laboratory-confirmed COVID-19 cases and 89,714 COVID-related deaths were reported [10]. Data on the clinical characteristic of COVID-19 patients in Poland are mostly limited to single-center studies [11,17,18]. A study carried out between 16 March and 7 April 2020 in one of the biggest COVID-19-dedicated hospitals showed that of the 169 patients hospitalized with COVID-19, 24.3% had developed ARDS [19]. This was the only available study on ARDS in patients with COVID-19 in Poland.

COVID-19 patients with ARDS often require additional IMV equipment and the involvement of specialized medical staff (e.g., anesthesiologists) [13]. Healthcare resources in the COVID-19 pandemic are significantly limited. Data on the prevalence of ARDS among COVID-19 patients, as well as factors associated with the development of ARDS are needed to inform decision-makers and healthcare professionals about resource allocation and healthcare capacity. Moreover, nationwide data on clinical characteristics of COVID-19 patients from multiple COVID-dedicated hospitals can be used for meta-analyses, clinical guidelines, and international comparisons of the effectiveness of anti-epidemic strategies in individual countries.

This study aimed to evaluate the prevalence of ARDS among patients hospitalized with COVID-19 in Poland as well as to characterize clinical outcomes in patients hospitalized with COVID-19-associated ARDS.

## 2. Materials and Methods

### 2.1. Data Source

The source of data for this study was hospital discharge reports on COVID-19 patients hospitalized in Poland between March and December 2020 (during the first and the second wave of the COVID-19 pandemic in Poland). Data were collected by the National Institute of Public Health National Institute of Hygiene—National Research Institute (NIPH NIH—NRI) as a part of the Nationwide General Hospital Morbidity Study—a branch of the Programme of Statistical Surveys of Official Statistics [20,21]. According to Polish law (ordinance of the Council of Ministers, Journal of Laws, item 2062) [21], all hospitals (both public and private, except the psychiatric facilities) are obligated to report hospitalization data (discharge reports) [20]. Anonymous data contained in the register are made available to scientists as part of public statistics on reasonable request (e.g., for research purposes).

Anonymous discharge reports are submitted to the Institute by public and private hospitals according to the dedicated template. The reporting obligation results from Polish law. Data on medical conditions are filled by the physicians based on the International Statistical Classification of Diseases and Related Health Problem (ICD-10) medical classification.

Discharge reports include data on gender, age, place of residence, hospital admission, discharge data, length of stay, the primary and secondary diagnosis, comorbidities, the outcome of hospitalization (death or survival).

### 2.2. COVID-19 Reporting

COVID-19 patients were identified through the ICD-10 discharge diagnosis codes: U07.1 (COVID-19, virus identified) or U07.2 (COVID-19, virus not identified). Code U07.1 is attributable to the laboratory-confirmed COVID-19 diagnosis. Code U7.02 is attributable to the clinical or epidemiological diagnosis of COVID-19. All patients with a medical diagnosis of COVID-19 (diagnosis codes: U07.1 or U7.02) were included in the analysis. A similar approach was applied in previous papers on hospitalized patients with COVID-19 in Poland, where the data source was medical registers [22,23,24].

### 2.3. ARDS Reporting, Comorbidities, and Outcomes

It was assumed that acute respiratory distress syndrome (ARDS) was the complication of COVID-19 since all the patients were admitted to the hospital with COVID-19 (U07.1 or U07.2) and the ARDS occurred within the same hospitalization. COVID-19 patients with COVID-19-associated ARDS were identified through the ICD-10 diagnosis code—J80 [25].

Comorbidities were grouped into categories based on the ICD-10 medical classification. The presence of the endocrine, nutritional and metabolic disease was based on the codes: E00-90. The presence of cardiovascular diseases was based on the codes: I00–I99. The presence of the disease of the genitourinary system was based on the code: N00-99. In addition to the whole disease groups, the incidence of selected common diseases whose impact on hospitalization outcomes have been reported in other studies was analyzed (COPD: J44; diabetes mellitus: E10–E14; chronic kidney disease: N18) [25].

Two different outcomes were analyzed: (1) incidence of ARDS among patients with COVID-19; and (2) in-hospital death (discharge status of hospitalization: death or survival) among patients with COVID-19-associated ARDS.

### 2.4. Ethics

This study was carried out following the principles expressed in the Declaration of Helsinki. Epidemiological reports were anonymous and available as part of public statistics. Patient consent was waived due to the fact that this study is a retrospective analysis and datasets used in this study were anonymous. The study protocol was reviewed and approved by the Ethics Review Board at the Central Clinical Hospital of the Ministry of the Interior and Administration in Warsaw, Warsaw, Poland (approval number 45/2021).

### 2.5. Statistical Analysis

The data were analyzed with SPSS version 28 (IBM, Armonk, NY, USA). The distribution of categorical variables was shown by counts and percentages. Statistical testing to compare categorical variables was completed using the independent samples chi-square test.

Associations between age, sex, and comorbidities with the development of ARDS were analyzed using logistic regression analyses. Moreover, logistic regression analyses were used to assess the associations between age, sex, and comorbidities with the outcome of hospitalization (in-hospital death). The development of ARDS and in-hospital death among patients hospitalized with COVID-19-associated ARDS were considered separately as a dependent variable in the model.

Age, sex, presence of at least one cardiovascular disease (I00–I99), COPD (J44), diabetes mellitus (E10–E14), and chronic kidney disease (N18) were considered as independent variables.

In univariate logistic regression analyses, all variables were considered separately. Multivariate logistic regression analyses included all the variables significantly associated with (1) the development of ARDS; and (2) the risk of in-hospital death.

The strength of association was measured by the odds ratio (OR) and 95% confidence intervals (CI). The level of statistical significance was set at 0.05.

## 3. Results

### 3.1. The Prevalence of ARDS among Patients Hospitalized with COVID-19

In 2020, a total of 116,539 patients were admitted to the hospital with COVID-19 (47.7% females). The characteristics of the study population have been published previously [19]. The prevalence of ARDS was 3.6%, 2.9% among females, and 4.4% among males (*p* < 0.001). The prevalence of ARDS increased with age (*p* < 0.001). The prevalence of cardiovascular diseases, endocrine, nutritional and metabolic diseases, diabetes mellitus, and COPD was significantly higher (*p* < 0.05) among patients with COVID-19-associated ARDS (Table 1).

### 3.2. Outcomes in Patients Hospitalized with COVID-19-Associated ARDS

Of the 4237 patients hospitalized with COVID-19-associated ARDS, 3764 deaths were reported (88.8%). The prevalence of in-hospital deaths increased with age (*p* < 0.001). The prevalence of cardiovascular diseases was higher among fatal cases compared to survivors (29.4% vs. 22.5%; *p* < 0.001). The prevalence of chronic kidney disease was higher among fatal cases (3.3%) compared to those who survived (2.7%; *p* = 0.02). Details are presented in Table 2.

### 3.3. Predictors of COVID-19-Associated ARDS

The results of the univariate and multivariate regression analyses are presented in Table 3. Several characteristics, such as age, sex, and presence of diabetes mellitus were significantly associated with the risk of COVID-19-associated ARDS (Table 3). Participants aged 60 years and over had more than three times higher odds of COVID-19-associated ARDS (Table 2). Men had higher odds of COVID-19-associated ARDS than women (OR = 1.55; 95% CI: 1.45–1.65; *p* < 0.001). Patients with COVID-19 and diabetes had higher odds of COVID-19-associated ARDS (OR = 1.16; 95% CI: 1.03–1.30; *p* = 0.01) compared to patients with COVID-19, but without diabetes (Table 3).

### 3.4. Predictors of Clinical Outcomes in Patients Hospitalized with COVID-19-Associated ARDS

In multivariate regression analyses, age and sex were significantly associated with the risk of in-hospital death among patients hospitalized with COVID-19-associated ARDS (Table 4). When compared with those aged under 60, patients aged 60–69 years had more than doubled the risk of in-hospital death (OR = 2.19; 95% CI: 1.72–2.79; *p* < 0.001). Patients aged 70–79 years had almost quadrupled risk of in-hospital death compared to those under 60 years of age (OR = 3.90; 95% CI: 2.99–5.10; *p* < 0.001). Over 80 years of age, the risk of in-hospital death increased nine times (OR = 9.00; 95% CI: 6.28–12.89; *p* < 0.001). Males had a higher risk of in-hospital death compared with females (OR = 1.27; 95% CI: 1.03–1.56; *p* = 0.02). Moreover, patients with cardiovascular diseases had a higher risk of in-hospital death compared with those without cardiovascular diseases (OR = 1.26; 95% CI: 1.00–1.59; *p* = 0.048). There was no significant impact of COPD, diabetes mellitus, and chronic kidney disease on the risk of in-hospital death among patients hospitalized with COVID-19-associated ARDS. Details are presented in Table 4.

## 4. Discussion

To the best of the author’s knowledge, this is the most comprehensive study on characteristics and clinical outcomes of patients hospitalized with COVID-19-associated ARDS in Poland. Data for this study was hospital discharge reports on more than 116 thousand COVID-19 patients hospitalized in Poland between March and December 2020. The results of this study show that 3.6% of patients hospitalized with COVID-19 had developed ARDS. The prevalence of in-hospital death among patients hospitalized with COVID-19-associated ARDS was 88.8%. This study showed that older age, male sex, and presence of diabetes were significantly associated with the risk of COVID-19-associated ARDS. Moreover, older age, male sex, and presence of cardiovascular diseases among patients with COVID-19-associated ARDS were significantly associated with the risk of in-hospital death.

Data included in this study were collected during the first two waves of the COVID-19 pandemic in Poland. During the first wave of the COVID-19 pandemic (the first half of 2020), numerous research centers and COVID-19-dedicated hospitals had published their own experiences on the clinical characteristics of COVID-19 patients [17,18,19]. Often these publications are based on data on the limited number of COVID-19 cases, so the prevalence of ARDS among COVID-19 patients may be overestimated [12,14].

Findings from the global literature survey on the incidence of ARDS and outcomes in patients hospitalized with COVID-19 between January and July 2020 showed that 33% had developed ARDS [12]. Wherein the prevalence of ARDS differed between individual regions of a given country [12]. For example, the prevalence of ARDS among patients hospitalized with COVID-19 in Italy varied from 19% in Brescia to 68% in Milan [12]. In Germany, almost half of patients hospitalized with COVID-19 had developed ARDS [12]. Findings from the only available study on ARDS among patients hospitalized with COVID-19 in Poland published by Nowak et al. showed that among 169 COVID-19 cases hospitalized between March and April 2020, almost a quarter had developed ARDS [19]. The prevalence of ARDS was 8.9% among survivors and 65.2% among non-survivors [19].

In this study, the prevalence of ARDS among patients hospitalized with COVID-19 was 3.6%. Differences between this study and previously published data may result from the fact that this study was based on hospital discharge reports on 116 thousand patients with COVID-19 who stayed in hospitals across the country. The COVID-19 treatment guidelines changed many times in 2020, which could have influenced the admission criteria and the number of patients who did not develop ARDS due to early treatment initiation [26]. However, we cannot exclude under-reporting of COVID-19-associated ARDS, especially in departments transformed into COVID-19-dedicated departments, which previously dealt with diseases other than the respiratory system (e.g., nephrology, surgery units).

Findings from the study of 10,815 ARDS cases in COVID-19 patients from seven countries showed that the overall pooled mortality estimate was 39% (95% CI: 0.23–0.56) [14]. The mortality estimates among COVID-19 patients with ARDS varied from 13% in Germany, 19% in France, 33% in Italy, 40% in Spain up to 73% in Poland [14]. In this study, 88.8% of patients hospitalized with COVID-19-associated ARDS died during hospitalization. The mortality rate presented in this study is higher, compared to previously reported by Nowak et al. [19]. Such a high mortality rate among patients hospitalized with COVID-19-associated ARDS may result from the high burden of COVID-19 in Poland during the second half of 2020. During the COVID-19, the capacity of the health care system in Poland was constantly increased. Temporary hospitals were established in sports facilities. Some general-profile hospitals were converted to COVID-19 dedicated hospitals. ARDS requires specialist care, including pulmonary physicians and anesthesiologists. We can hypothesize that a higher mortality rate was observed in district hospitals, where there were no lung disease departments or intensive care units. Moreover, we can hypothesize that the high mortality rate presented in this study may result from nosocomial infections [27]. Nosocomial clusters play an important role in SARS-CoV-2 transmission. Recent international scientific data on prevention and protection measures all over the world showed that correct management of healthcare workers’ contacts is essential to avoid nosocomial clusters [28]. The implementation of a protocol for protecting the safety of healthcare workers in the course of their daily work is necessary to control the outbreak throughout society [29]. Vimercati et al. showed that the correct use of personal protective equipment and the early identification of symptomatic workers are essential factors to avoiding nosocomial clusters [28,30]. Moreover, findings from the prospective observational study in Italy [31] showed that integrated hospital infection control strategy, consisting of dedicated areas for COVID-19 patients, strict measures for personal protective equipment use, and mass surveillance are effective tools to prevent SARS-CoV-2 infection among healthcare workers.

Previous studies showed that older age, male sex, and presence of comorbidities are associated with severe course of COVID 19 [1,3,22,23,24]. However, there is limited data primarily focused on the factors associated with the development of ARDS among patients hospitalized with COVID-19, as well as the risk of in-hospital death among patients with COVID-19-associated ARDS. This study confirmed that older age and male sex are the most important factors associated with the risk of COVID-19-associated ARDS. Moreover, the presence of diabetes was significantly associated with the risk of COVID-19-associated ARDS. The impact of diabetes on the severity of COVID-19 requires further investigation. In this study, older age, male sex, and presence of cardiovascular diseases among patients with COVID-19-associated ARDS were significantly associated with the risk of in-hospital death. This observation is in line with the previous studies [22,23]. It is estimated that one-third of deaths among patients with COVID-19-associated ARDS is due to respiratory failure combined with cardiac failure [32].

This study has several practical implications. This is the first clinical characteristic of patients with COVID-19-associated ARSD in Poland. Moreover, factors associated with the development of ARDS in COVID-19 patients, as well as factors associated with the risk of in-hospital death among patients with COVID-19-associated ARDS were described. Clinicians may use the above-mentioned data to evaluate and update clinical guidelines on the management of COVID-19. Moreover, identification of the groups with the higher risk of COVID-19-associated ARDS and in-hospital death may be used by health managers to estimate the need for healthcare during the COVID-19 pandemic and to better allocate resources.

This study has several limitations. First, this study is a retrospective, secondary analysis of epidemiological data that are reported using one specific template. Data are limited to those available in the hospital discharge report template. Data on the severity of ARDS were not available. Secondly, the format of discharge reports, as well as the data quality, does not allow the identification of COVID patients receiving invasive mechanical ventilation (IMV). Thirdly, COVID-19 treatment (e.g., the use of corticosteroids) and its outcomes were not assessed. Moreover, there are other important factors such as obesity and admission to ICU factors that could affect the results, but due to the format of discharge reports, the above-mentioned data were not included in the analysis. Nevertheless, this is the first nationwide study on the COVID-19-associated ARDS among patients hospitalized with COVID-19 in Poland in 2020.

## 5. Conclusions

Among patients hospitalized with COVID-19 in Poland, the prevalence of ARDS was relatively low. However, the in-hospital mortality rate in patients with COVID-19-associated ARDS was higher compared to other EU countries. Individuals over 60 years of age, males, as well as those with diabetes, are at higher risk of development of COVID-19-associated ARDS. Older age, male sex, and presence of at least one cardiovascular disease were associated with the higher risk of in-hospital death among patients with COVID-19 associated ARDS.

## Figures and Tables

**Table 1:** The prevalence of ARDS among patients hospitalized with COVID-19, Poland, March–December 2020.

Variable	Total Sample	ARDS	Non-ARDS	*p*
	*n* (%)	*n* (%)	*n* (%)	
*n* (%)	116,539 (100.0%)	4237 (3.6%)	112,302 (96.4%)	
Sex				
women	55,624 (47.7%)	1587 (37.5%)	54,037 (48.1%)	<0.001
men	60,915 (52.3%)	2650 (62.5%)	58,265 (51.9%)	
Age (years)				
0–9	3725 (3.2%)	0 (0.0%)	3725 (3.3%)	<0.001
10–19	2154 (1.8%)	1 (0.0%)	2153 (1.9%)	
20–29	5710 (4.9%)	10 (0.2%)	5700 (5.1%)	
30–39	8794 (7.5%)	58 (0.7%)	8736 (7.8%)	
40–49	11,147 (9.6%)	178 (4.2%)	10,969 (9.8%)	
50–59	15,248 (13.1%)	460 (10.9%)	14,788 (13.2%)	
60–69	24,358 (20.9%)	1143 (27.0%)	23,215 (20.7%)	
70–79	23,678 (20.3%)	1284 (30.3%)	22,394 (19.9%)	
80+	21,725 (18.6%)	1103 (26.0%)	20,622 (18.4%)	
Presence of at least one cardiovascular disease (I00–I99)				
Yes	26,541 (22.8%)	1246 (29.4%)	25,295 (22.5%)	<0.001
Presence of at least one endocrine, nutritional and metabolic disease (E00-99)				
Yes	10,165 (8.7%)	443 (10.5%)	9722 (8.7%)	<0.01
Presence of at least one disease of the genitourinary system (N00-99)				
Yes	6280 (5.4%)	242 (5.7%)	6038 (5.4%)	0.3
COPD				
Yes	1324 (1.1%)	70 (1.7%)	1254 (1.1%)	0.001
Arterial hypertension				
Yes	12,227 (10.5%)	425 (10.0%)	11,802 (10.5%)	0.3
Diabetes mellitus				
Yes	6924 (5.9%)	349 (8.2%)	6575 (5.9%)	<0.001
Chronic kidney disease				
Yes	3139 (2.7%)	138 (3.3%)	3001 (2.7%)	0.02

**Table 2:** The prevalence of in-hospital death among patients hospitalized with COVID-19-associated ARDS, Poland, March–December 2020.

Variable	Patients Hospitalized with COVID-19-Associated ARDS *n* = 4237		*p*
	Fatal Cases *n* (%)	Non-Fatal Cases *n* (%)	
*n* (%)	3764 (88.8%)	473 (11.2%)	
Sex			
women	1408 (37.4%)	179 (37.8%)	0.8
men	2356 (62.6%)	294 (62.2%)	
Age (years)			
0–9	0 (0.0%)	0 (0.0%)	<0.001
10–19	1 (0.0%)	0 (0.0%)	
20–29	8 (0.2%)	2 (0.4%)	
30–39	37 (1.0%)	21 (4.4%)	
40–49	113 (3.0%)	65 (13.7%)	
50–59	370 (9.8%)	90 (19.0%)	
60–69	922 (26.4%)	151 (31.9%)	
70–79	1181 (31.4%)	103 (21.8%)	
80+	1062 (28.2%)	41 (8.7%)	
Presence of at least one cardiovascular disease (I00–I99)			
Yes	1136 (30.2%)	110 (23.3%)	0.002
Presence of at least one endocrine, nutritional and metabolic disease (E00-99)			
Yes	387 (10.3%)	56 (11.8%)	0.3
Presence of at least one disease of the genitourinary system (N00-99)			
Yes	221 (5.9%)	21 (4.4%)	0.2
COPD			
Yes	64 (1.7%)	6 (1.3%)	0.5
Arterial hypertension			
Yes	359 (9.5%)	66 (14.0%)	0.003
Diabetes mellitus			
Yes	311 (8.3%)	38 (8.0%)	0.9
Chronic kidney disease			
Yes	127 (3.4%)	11 (2.3%)	0.2

**Table 3:** Odds ratios (OR) and 95% confidence intervals (CI) for the acute respiratory distress syndrome (ARDS) in a group of 116,539 patients hospitalized with COVID-19—Poland, March–December 2020.

	The Incidence of Acute Respiratory Distress Syndrome (ARDS)					
Variable	Univariate Logistic Regression			Multivariate Logistic Regression ^a^		
	OR	95% CI	*p*	OR	95% CI	*p*
Age (years)						
<60	1.00	Reference		1.00	Reference	
60–69	3.21	2.92–3.53	<0.001	3.07	2.79–3.38	<0.001
70–79	3.74	3.40–4.10	<0.001	3.65	3.32–4.01	<0.001
≥80	3.49	3.17–3.84	<0.001	3.58	3.24–3.94	<0.001
Sex						
women	1.00	Reference		1.00	Reference	
men	1.55	1.45–1.65	<0.001	1.55	1.45–1.65	<0.001
Presence of at least one cardiovascular disease (I00–I99)						
No	1.00	Reference		1.00	Reference	
Yes	1.43	1.34–1.53	<0.001	1.02	0.95–1.09	0.2
COPD						
No	1.00	Reference		1.00	Reference	
Yes	1.49	1.17–1.90	0.001	1.01	0.79–1.29	0.9
Diabetes mellitus						
No	1.00	Reference		1.00	Reference	
Yes	1.44	1.29–1.62	<0.001	1.16	1.03–1.30	0.01
Chronic kidney disease						
No	1.00	Reference		1.00	Reference	
Yes	1.23	1.03–1.46	0.02	0.92	0.78–1.10	0.4

^a^ Fully adjusted model including all statistically significant characteristics in the univariable analyses.

**Table 4:** Odds ratios (OR) and 95% confidence intervals (CI) for in-hospital death in a group of 4237 patients hospitalized with COVID-19-associated ARDS—Poland, March–December 2020.

	In-Hospital Death in a Group of 4237 Patients Hospitalized with COVID-19-Associated ARDS					
Variable	Univariate Logistic Regression			Multivariate Logistic Regression ^a^		
	OR	95% CI	*p*	OR	95% CI	*p*
Age (years)						
<60	1.00	Reference		1.00	Reference	
60–69	2.21	1.74–2.81	<0.001	2.19	1.72–2.78	<0.001
70–79	3.86	2.97–5.02	<0.001	3.89	2.98–5.07	<0.001
≥80	8.72	6.11–12.43	<0.001	8.94	6.24–12.80	<0.001
Sex						
women	1.00	Reference		1.00	Reference	
men	1.02	0.84–1.24	0.9	1.27	1.03–1.56	0.02
Presence of at least one cardiovascular disease (I00–I99)						
No	1.00	Reference		1.00	Reference	
Yes	1.43	1.14–1.79	0.002	1.26	1.00–1.59	0.048
COPD						
No	1.00	Reference			-	
Yes	1.35	0.58–3.13	0.5			
Diabetes mellitus						
No	1.00	Reference			-	
Yes	1.03	0.73–1.47	0.9			
Chronic kidney disease						
No	1.00	Reference			-	
Yes	1.47	0.79–2.74	0.2			

^a^ Fully adjusted model including all statistically significant characteristics in the univariable analyses and gender.

## Data Availability

The datasets generated during and/or analyzed during the current study are available from the corresponding author on reasonable request.
